# An Epidemiological Study of Sleep-Related Disorders and Their Association With Cardiovascular Risk Factors in an Adult Population (30-69 Years) of South Delhi

**DOI:** 10.7759/cureus.113932

**Published:** 2026-08-04

**Authors:** Vishal K Singh, Farzana Islam, Rambha Pathak, Aqsa Shaikh, Srikrishna Sulgodu Ramachandra

**Affiliations:** 1 Community Medicine, Narayan Medical College and Hospital, Sasaram, IND; 2 Adverse Events Following Immunization, Ministry of Health and Family Welfare, New Delhi, IND; 3 Community Medicine, Hamdard Institute of Medical Sciences and Research (HIMSR), New Delhi, IND; 4 Community Medicine, Government Institute of Medical Sciences, Greater Noida, Greater Noida, IND; 5 Community Medicine, Jamia Hamdard, New Delhi, IND; 6 Community Medicine, Public Health, Narayan Medical College and Hospital, Sasaram, IND

**Keywords:** cardiometabolic risk, ess, excessive daytime sleepiness, gsaq, insomnia, obstructive sleep apnea, psqi, sleep-related disorders, south delhi, stop-bang

## Abstract

Background

Sleep-related disorders (SRDs) are a major yet underdiagnosed public health burden in India, and community-level evidence linking them with cardiometabolic risk factors remains scarce. This study aimed to estimate the point prevalence of selected SRDs assessed by four validated screening questionnaires, and to examine their association with cardiovascular risk factors in an urban adult population of South Delhi.

Methodology

A community-based, cross-sectional study was conducted among 700 apparently healthy adults aged 30-69 years recruited by four-stage random sampling from five wards of the Central Zone of the South Delhi Municipal Corporation (SDMC). SRDs were assessed using the Global Sleep Assessment Questionnaire (GSAQ), Pittsburgh Sleep Quality Index (PSQI), Epworth Sleepiness Scale (ESS), and STOP-Bang questionnaire. Hypertension was defined as blood pressure ≥140/90 mmHg and diabetes as fasting blood glucose ≥126 mg/dL. Associations and independent predictors were identified using the chi-square test and binary logistic regression in SPSS Statistics version 26 (IBM Corp., Armonk, NY, USA).

Results

Sleep-related breathing disorder (SRBD) prevalence was 37.0% and primary insomnia 36.0% by GSAQ. Daytime sleepiness (ESS ≥8) was present in 13.3%, obstructive sleep apnea (OSA; STOP-Bang ≥3) in 20.9%, and poor sleep quality (PSQI >5) in 23.9%. OSA was significantly more prevalent in males (41.5%) than females (13.9%) (p < 0.001). Newly detected hypertension was 19.3% and diabetes 12.3%. In multivariable binary logistic regression across seven sleep outcomes, restless legs syndrome was independently associated with female sex (adjusted odds ratio (AOR) = 4.05) and physical inactivity (AOR = 2.61); daytime sleepiness with diabetes (AOR = 1.84) and abnormal waist-hip ratio (AOR = 1.62); and insomnia with mental disorder (AOR = 2.28) and poor sleep latency (AOR = 1.77) with female sex. Because male sex, body mass index, and blood pressure are themselves STOP-Bang scoring components, the estimates obtained for STOP-Bang screen positivity are structural associations with the instrument rather than independent predictors of OSA, and are reported as such.

Conclusions

Screening positivity for SRDs was high in this community. STOP-Bang screen positivity was concentrated among men and older participants, whereas GSAQ-defined SRBD was more frequent in women and not explained by anthropometric factors, suggesting the two constructs capture different phenomena. Questionnaire-based sleep screening is inexpensive and scalable and could be integrated into existing non-communicable disease screening encounters. As this was a cross-sectional screening survey, no inference is drawn regarding the effect of any intervention on these outcomes.

## Introduction

Sleep is a fundamental physiological process essential for physical restoration, metabolic regulation, cognitive consolidation, and psychological well-being. Disruptions to this process, collectively termed sleep-related disorders (SRDs), constitute one of the most prevalent yet consistently underrecognized categories of chronic disease. The International Statistical Classification of Diseases and Related Health Problems, 10th revision (ICD-10) classifies sleep disorders principally under disorders of the nervous system (category G47), encompassing disorders of initiating and maintaining sleep (insomnias), disorders of excessive somnolence (hypersomnias), disorders of the sleep-wake schedule, sleep apnea, and narcolepsy and cataplexy, together with the non-organic sleep disorders of the mental and behavioral chapter (category F51), which include non-organic insomnia, non-organic hypersomnia, sleepwalking, sleep terrors, and nightmares [1]. These categories broadly correspond to the symptom-based domains of insomnia, sleep-related breathing disorders (SRBDs), hypersomnolence, circadian rhythm disorders, parasomnias, and sleep-related movement disorders recognized in the specialist sleep literature [2].

Globally, an estimated 20-30% of adults experience SRDs of clinically meaningful severity, yet formal diagnosis is sought in only 6-15% of affected individuals, reflecting a diagnostic gap driven by poor awareness, symptom normalization, and limited access to specialist services [3]. In India, population-based studies report SRD prevalence ranging from 20% to 55% across urban and rural populations [4,5]. Suri et al. reported an SRD prevalence of 55% in urban Delhi, whereas Panda et al. found rates of 20-34% in South India using standardized questionnaire-based assessment [4,5].

Among specific SRDs, obstructive sleep apnea (OSA) is defined by the ICD-10 as a disorder characterized by recurrent apnea during sleep despite persistent respiratory efforts, associated with cardiac arrhythmias and elevation of both systemic and pulmonary arterial pressures [1]. Its pathophysiology involves anatomical narrowing of the upper airway, exacerbated by obesity, craniofacial abnormalities, and neuromuscular laxity, resulting in repetitive cycles of hypoxia, hypercapnia, arousal, and sympathetic activation. Community-based estimates in India place OSA prevalence at approximately 9-13.7% [6,7]. OSA disproportionately affects middle-aged males and individuals with obesity and is an established independent risk factor for hypertension, cardiac arrhythmia, coronary artery disease, and type 2 diabetes mellitus [8].

Excessive daytime sleepiness (EDS) impairs occupational performance, drives road traffic accidents, and frequently signals an underlying undiagnosed sleep disorder such as OSA or narcolepsy [9]. Population estimates of EDS in India range from 20% to 48%, with obesity being the most consistently identified modifiable risk factor [10].

Insomnia is reported as a symptom by approximately one-third of adults, with 6-15% meeting the criteria for a formal disorder [3]. It is strongly associated with depression and anxiety and is increasingly recognized as a bidirectional risk factor for metabolic and cardiovascular disease [11].

The relationship between SRDs and cardiometabolic risk is complex and bidirectional. Sleep-disordered breathing drives sympathetic nervous system activation and endothelial dysfunction, elevating blood pressure and accelerating atherosclerosis [12]. Intermittent nocturnal hypoxia impairs glucose metabolism and promotes insulin resistance [13]. Conversely, hypertension and diabetes alter sleep architecture and worsen nocturnal respiratory function, creating a self-perpetuating cycle [14]. Central obesity narrows the upper airway and reduces functional residual capacity, establishing body mass index (BMI) and waist-hip ratio as the most powerful modifiable predictors of SRBD [15]. Chronic sleep deprivation additionally produces measurable cardiovascular, inflammatory, and metabolic consequences [16].

Despite this evidence, community-based epidemiological data on the full SRD spectrum and its cardiometabolic associations in India remain limited. Most published studies are hospital-based, restricting generalizability. Polysomnography, the diagnostic gold standard, requires laboratory conditions and trained personnel, making it impractical for community surveillance. Validated questionnaire-based tools provide a sensitive, inexpensive, and scalable alternative, achieving sensitivities of 80-93% for key sleep disorders [17,18].

Physical inactivity is an additional determinant of SRDs. The World Health Organization (WHO) recommends at least 600 metabolic equivalent of task (MET)-minutes per week of moderate-to-vigorous physical activity; sedentary behavior worsens sleep quality, increases OSA risk, and amplifies the metabolic consequences of sleep disruption [19].

This study was undertaken to estimate the point prevalence of selected SRDs, assessed by four validated screening questionnaires, in an urban adult population of South Delhi, to examine their association with sociodemographic characteristics and cardiometabolic risk factors, to identify independent predictors of SRDs using multivariable logistic regression, and to present sex-stratified prevalence for all major outcomes.

## Materials and methods

Study design, area, and duration

A community-based, cross-sectional, epidemiological study was conducted in South Delhi, as defined by the South Delhi Municipal Corporation (SDMC). The SDMC serves approximately 5.6 million citizens across 656.91 km², subdivided into four administrative zones comprising 104 wards. The study spanned from November 2019 to March 2021 (17 months). This period comprised ethical clearance, protocol finalization, and instrument permissions, followed by the suspension imposed by the COVID-19 national lockdown; the field phase itself was completed within approximately four months. No data were collected before or during the lockdown. Recruitment began only after restrictions were lifted, and all participants were therefore enrolled within a single, continuous post-lockdown window. Comparison of pre- and post-lockdown recruitment periods is consequently not applicable to these data, and no calendar-period effect of the lockdown on the estimates can arise. Each participant was assessed on a single occasion during one household visit, at which the questionnaires, anthropometry, blood pressure measurement, and blood glucose estimation were all completed; no participant was assessed more than once. All COVID-19-appropriate protocols, including masks, hand sanitizers, and interviewer vaccination, were observed throughout data collection.

Study population and eligibility

Apparently healthy adults aged 30-69 years residing in the study area for at least six months were eligible. Individuals with known hypertension, diabetes mellitus, thyroid disorders, cardiovascular disease, serious medical illness, or surgery within the preceding six months were excluded, as were pregnant and lactating women.

Eligibility was established by self-reported history only, using the screening question placed at the beginning of the study proforma (Appendix). Participants were asked whether they had ever been diagnosed by a physician with hypertension, diabetes mellitus, thyroid disease, cardiovascular disease, or any other chronic illness, or had undergone surgery in the preceding six months. If the answer to any of these was affirmative, the interview was terminated at that point, and no further data were collected; only participants reporting no such history proceeded to the remainder of the questionnaire and to physical and biochemical assessment. No medical records, prescriptions, or laboratory reports were examined to corroborate the reported history, and this is acknowledged as a limitation. This design ensured that all cardiometabolic conditions detected during the study represented previously undiagnosed findings in participants who considered themselves healthy.

Sample size calculation

Sample size was calculated using n = Z²P(1-P)/e², where Z = 1.96 (95% confidence), P = 0.20 (expected SRD prevalence from prior Indian literature) [4], and e = 15% relative error, yielding a minimum of 683. After adding 20% for non-response, 820 individuals were approached; 700 gave written informed consent and constituted the final analytic sample.

Multistage random sampling procedure

**Figure 1:**
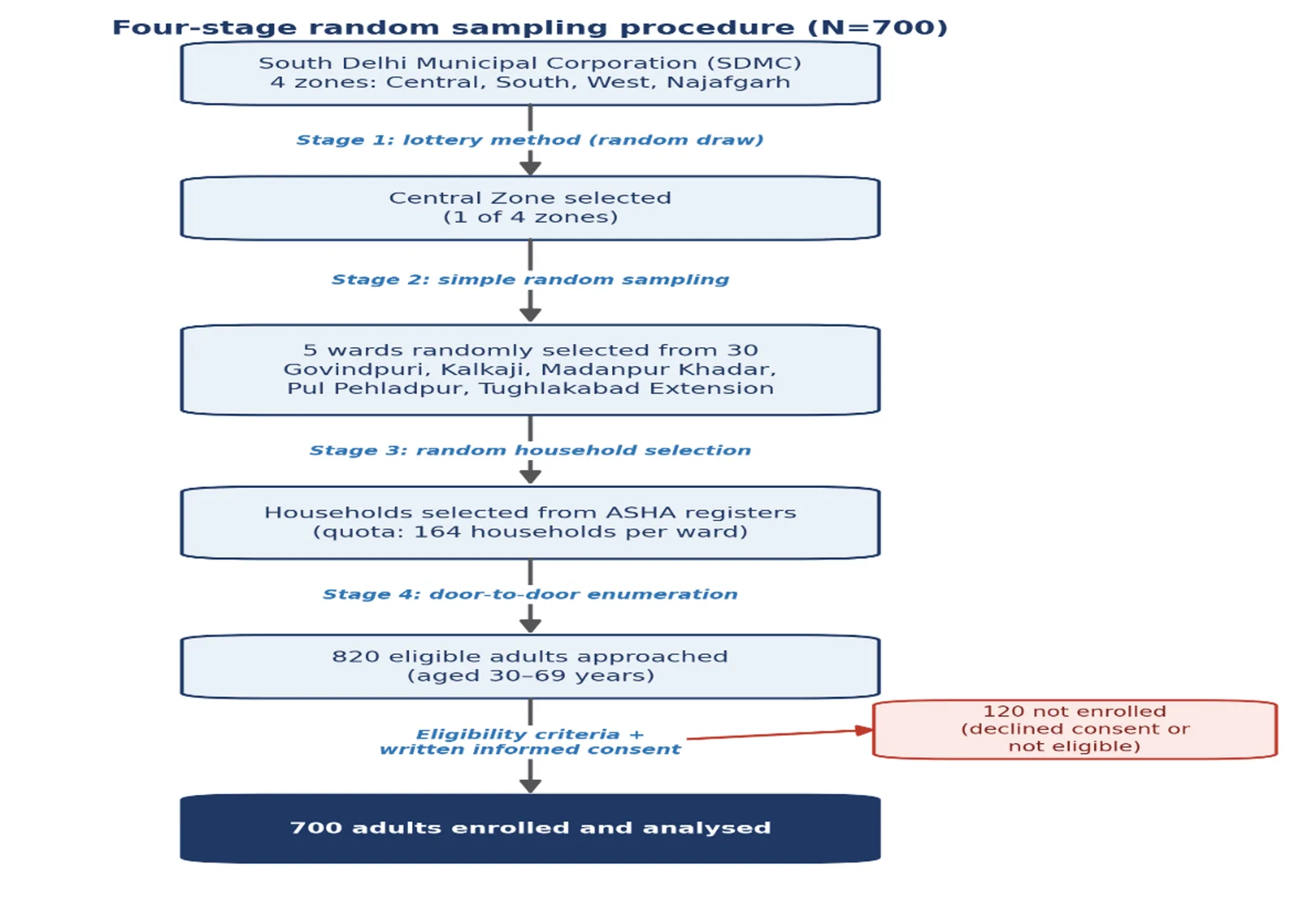
Four-stage random sampling procedure (N = 700). Four-stage random sampling procedure (N = 700) in the South Delhi Municipal Corporation (SDMC) from four zones: Central, South, West, Najafgarh → Stage 1: lottery method (random draw) → Central Zone selected (one of four zones) → Stage 2: simple random sampling → five wards randomly selected from 30 — Govindpuri, Kalkaji, Madanpur Khadar, Pul Pehladpur, Tughlakabad Extension → Stage 3: random household selection → Households selected from ASHA registers (quota: 164 households per ward) → Stage 4: door-to-door enumeration → 820 eligible adults approached (aged 30–69 years) → Eligibility criteria + written informed consent → 700 adults enrolled and analysed (branch: 120 not enrolled: declined consent or not eligible).

A four-stage probability-based sampling strategy was employed, as shown in Figure 1, following the stratified multistage design used in the National Family Health Survey. At the first stage, the four administrative zones of the SDMC (Central, South, West, and Najafgarh) were listed and one zone, the Central Zone, was selected at random by lottery. At the second stage, all 30 wards of the Central Zone were listed, and five wards, namely, Govindpuri, Kalkaji, Madanpur Khadar, Pul Pehladpur, and Tughlakabad Extension, were selected by simple random sampling; the number of wards to be sampled was itself determined at random. At the third stage, households were selected at random from the ASHA registers of each selected ward, with an equal quota of 164 households per ward. At the fourth stage, door-to-door enumeration was performed, and one eligible adult was enrolled per household, so that no household contributed more than one participant. Of 820 eligible adults approached, 700 gave written informed consent and were enrolled.

Study variables

The dependent and independent variables analyzed are summarised in Table 1.

**Table 1:** Study variables. GSAQ = Global Sleep Assessment Questionnaire; ESS = Epworth Sleepiness Scale; PSQI = Pittsburgh Sleep Quality Index; WHO = World Health Organization; GPAQ = Global Physical Activity Questionnaire; MET = metabolic equivalent of task; JNC = Joint National Committee

Type	Variable	Operational definition/Source
Dependent variables	Primary insomnia	GSAQ Q1, positive if response other than “Never”
	Insomnia with mental disorder	GSAQ Q1 + Q11
	Sleep-related breathing disorder	GSAQ Q5 + Q6
	Restless leg syndrome	GSAQ Q7
	Periodic limb movement	GSAQ Q8
	Parasomnia	GSAQ Q9
	Daytime sleepiness	ESS score ≥8
	Obstructive sleep apnea risk	STOP-Bang score ≥3
	Sleep quality and its seven components	PSQI; global score >5 indicates poor sleep quality
Independent variables	Age	Completed years, grouped 30–40, 41–50, 51–60, 61–69
	Sex	Male/Female
	Education	Literate/Illiterate
	Religion	Hindu/Muslim/other
	Socioeconomic status	Modified Kuppuswamy scale 2021 [20]
	Body mass index	Weight (kg)/height (m)²; WHO classification [21]
	Waist-hip ratio	Abnormal if ≥0.90 (men) or ≥0.85 (women), WHO criteria
	Neck circumference	Measured in cm; STOP-Bang component
	Mallampati score	Class I-IV on oropharyngeal examination
	Physical activity	GPAQ; sufficient if ≥600 MET-minutes per week
	Blood pressure	Hypertension if ≥140/90 mmHg (JNC) [22,23]
	Blood glucose	Diabetes if fasting blood glucose ≥126 mg/dL [24,25]

Detection of hypertension and diabetes

As all participants with known hypertension and diabetes were excluded at enrolment, cases detected during the study represented newly detected (previously undiagnosed) disease. The detection methodology is summarized in Table 2.

**Table 2:** Methodology for the detection of hypertension and diabetes. JNC = Joint National Committee; FBG = fasting blood glucose; BP = blood pressure; ICMR = Indian Council of Medical Research; NIDDK = National Institute of Diabetes and Digestive and Kidney Diseases

Parameter	Hypertension	Diabetes mellitus
Measurement instrument	Digital sphygmomanometer	Digital glucometer
Measurement site/sample	Left arm, sitting	Capillary fingerprick
Blood sample volume	Not applicable	0.1 ml
Preparation	Seated rest ≥5 minutes, back supported, feet flat, arm at the heart level; caffeine, exercise, and smoking avoided	Fasting ≥8 hours without caloric intake before the fasting sample
Cuff	Bladder encircling ≥80% of the arm (JNC 7)	Not applicable
Readings taken	Three at 5-minute intervals in one sitting	Step 1: random blood glucose in all participants. Step 2: if random glucose ≥200 mg/dL, FBG the following morning
Value used for analysis	Mean of last two readings	FBG
Diagnostic threshold	BP ≥140/90 mmHg (JNC 8) [23]	FBG ≥126 mg/dL (ICMR 2018 [24]; NIDDK [25])

Blood Pressure

Measurement followed the technique recommended in the Seventh Report of the Joint National Committee (JNC 7) [22]. Participants were seated quietly for at least five minutes before the first reading, with the back supported, feet flat on the floor, and the arm supported at heart level, having avoided caffeine, exercise, and smoking beforehand. A cuff of appropriate size was used, the bladder encircling at least 80% of the arm, as JNC 7 specifies no single standard cuff dimension. Three readings were taken at five-minute intervals in the same sitting using a validated digital sphygmomanometer, and the mean of the last two readings was used for analysis. The diagnostic threshold of ≥140/90 mmHg follows the Eighth Joint National Committee (JNC 8) classification [23].

Blood Glucose

Estimation was performed in two steps. Random blood glucose was measured at the household visit using a digital glucometer. Where the random value was ≥200 mg/dL, the participant was revisited the following morning and a fasting blood glucose was measured after at least eight hours without caloric intake. A fasting blood glucose ≥126 mg/dL was taken as diagnostic of diabetes, in accordance with the Indian Council of Medical Research (ICMR) 2018 criteria [24], which apply the same threshold as the National Institute of Diabetes and Digestive and Kidney Diseases (NIDDK) [25].

Equipment

A digital weighing machine for adults, a non-stretchable measuring tape, a digital blood pressure machine, and a digital glucometer were used. All equipment was validated by the Biomedical Engineering Department at Hakeem Abdul Hameed Centenary Hospital (HAHC), New Delhi, before data collection commenced. All newly detected cases were counselled and referred for tertiary management.

Study tools and scoring criteria

Six pre-tested, validated instruments were administered. Prior written permission to use and translate each instrument was obtained from the respective copyright holders before data collection commenced.

All instruments were interviewer-administered in Hindi. Every item was read aloud to the participant in Hindi by the interviewer, and the response was recorded by the interviewer. No questionnaire was self-completed, and no participant was required to read any instrument. Forward and back translation into Hindi was performed only for those instruments for which no validated Hindi version existed, namely, the Global Sleep Assessment Questionnaire (GSAQ), the Pittsburgh Sleep Quality Index (PSQI), and the Global Physical Activity Questionnaire (GPAQ). A validated Hindi version was available and used for the Epworth Sleepiness Scale (ESS) [26]. For the STOP-Bang questionnaire, no Hindi version was available to the authors, and the pre-validated Urdu version was therefore used as the source of item wording; as Hindi and Urdu are mutually intelligible in speech, these eight items were also delivered orally in Hindi. Dr Aqsa Shaikh, co-author of this study and literate in Urdu, assisted the first author in reading and rendering the eight Urdu items so that the spoken Hindi delivery corresponded faithfully to the validated source.

The instruments were pre-tested on 25 adults to assess feasibility in the study population. Internal consistency was assessed in this pre-test sample using Cronbach’s alpha computed from the item variances. For the seven PSQI component scores, alpha was 0.70, indicating acceptable internal consistency. Cronbach’s alpha was not computed for the ESS, STOP-Bang, or GPAQ, for which only total scores were retained, nor for the GSAQ, whose six sub-scores assess distinct and conceptually independent sleep disorders rather than a single underlying construct, so that a coefficient of internal consistency would not be interpretable.

The GSAQ [17] is an 11-item instrument assessing six sleep disorders over the past four weeks, scored as Never, Sometimes, Usually, or Always. Any response other than “Never” is considered positive for the assigned disorder. Sensitivity and specificity, respectively, are as follows: primary insomnia (Q1; 79% and 57%), insomnia with mental disorder (Q1+Q11; 83% and 51%), SRBD (Q5+Q6; 93% and 58%), restless leg syndrome (Q7; 90% and 80%), periodic limb movement (Q8; 93% and 52%), and parasomnia (Q9; 100% and 49%). The GSAQ permits multiple positive disorder responses per participant.

The PSQI [18] assesses the following seven components of sleep quality over the past month: subjective sleep quality, sleep latency, sleep duration, habitual sleep efficiency, sleep disturbances, sleep medication use, and daytime dysfunction. The global PSQI score ranges from 0 to 21; a global score >5, that is, a score of 6 or more, indicates poor sleep quality (sensitivity, 89.6%; specificity, 86.5%). This threshold was applied consistently throughout the analysis, and participants scoring exactly 5 (n = 68) were classified as good sleepers.

The ESS [27,26] is an eight-item scale rating the likelihood of dozing in everyday situations (0-3 per item; total = 0-24). Two distinct scoring conventions attach to this instrument, and both are used here. The first is the descriptive severity classification published with the original scale by Johns [27], which grades the total score into the following five tiers: lower normal (0-5), higher normal (6-10), mild excessive (11-12), moderate excessive (13-15), and severe excessive (16-24). The second is the diagnostic cut-off derived subsequently by Bajpai et al. in the validation of the Hindi version among a North Indian population [26], who reported a cut-off of ≥8 for the identification of daytime sleepiness. Because the Hindi version was the instrument administered in this study, the ≥8 cut-off from its validation was adopted as the case definition for daytime sleepiness, while the five-tier classification of Johns is reported separately as a description of severity. The two therefore serve different purposes and are not alternative definitions of the same quantity.

The STOP-Bang questionnaire [28,29] comprises eight yes/no items assessing snoring, tiredness, observed apnea, blood pressure, BMI, age, neck circumference, and gender. Each “yes” scores one point; scores of 0-2 indicate low OSA risk, 3-4 intermediate risk, and 5-8 high risk. A cut-off of ≥3 was used to define OSA positivity (sensitivity, 84%). The pre-validated Urdu version was used as the source of item wording, administered orally in Hindi as described above.

The GPAQ [30] is a 16-item WHO-developed tool assessing physical activity across three domains (work, travel, and recreation). Data are expressed in MET-minutes per week; ≥600 MET-minutes per week os defined as sufficient physical activity.

Data collection

All data were collected at the participant’s home during door-to-door enumeration, between 09:00 and 16:00. The study formed the MD thesis of the corresponding author, who personally administered all questionnaires and performed all anthropometric, clinical, and blood pressure measurements; assistance from colleagues trained in social work was confined to blood glucose estimation. Because a single interviewer conducted every interview, inter-interviewer variation does not arise in this study. Data were captured directly on Epicollect5 software [31] using a mobile device, with range and logic checks applied at the point of entry, eliminating a manual transcription step. Data cleaning and statistical analysis were also performed by the corresponding author.

Statistical analysis

Data were entered in Microsoft Excel (Microsoft Corp., Redmond, WA, USA) and analyzed using SPSS Statistics version 26 (IBM Corp., Armonk, NY, USA). Descriptive statistics (frequencies, percentages, means, and standard deviations) were computed, and sex-stratified prevalence was reported for all major SRD and cardiometabolic outcomes. Bivariate associations were examined using the Pearson chi-square test; the Fisher exact test was applied where expected cell counts were below five. For every bivariate comparison, the chi-square test statistic (χ²), its degrees of freedom (df), and the p-value are reported. Variables showing a statistically significant association with a given outcome on bivariate chi-square testing were then entered together into a binary logistic regression model for that outcome, so that the multivariable analysis was restricted to variables for which a prior association had been demonstrated. Results are expressed as adjusted odds ratios (AORs) with 95% confidence intervals (CI); the corresponding B coefficient, its standard error, the Wald χ² statistic (df = 1) and the p-value are reported for each coefficient, and the Nagelkerke R² and the Hosmer-Lemeshow goodness-of-fit statistic are reported for each model in the text. A p-value <0.05 was considered statistically significant.

Ethical considerations

Ethical approval was obtained from the Jamia Hamdard Institutional Ethics Committee (approval number: 15/19; meeting held on 26/11/2019). A participant information sheet was provided to every participant, and the study was explained in Hindi before enrolment. Literate participants read the information sheet and signed the consent form. For illiterate participants, the information sheet and consent form were read aloud in Hindi by the investigator, questions were invited and answered, and consent was recorded by the participant’s thumb impression, witnessed and countersigned by a literate household member whose signature appears on the consent form. Consent preceded all data collection and covered questionnaire administration, physical measurement, capillary blood sampling, and data storage. Data were stored securely for five years in accordance with institutional policy.

## Results

Sociodemographic profile

A total of 700 participants were enrolled. Females constituted 74.9% (n = 524) and males 25.1% (n = 176). The higher female proportion reflects daytime data collection in a working-age community, where male workforce participation in urban Delhi exceeds 80%, leaving men largely unavailable during household-visiting hours. The mean age was 39.41 ± 9.2 years; 64.4% (n = 451) were aged 30-40 years, 21.1% (n = 148) were aged 41-50 years, 9.7% (n = 68) were aged 51-60 years, and 4.8% (n = 33) were aged 61-69 years. Most participants (92.4%) were currently married. By religion, 71.3% were Hindu and 27.7% were Muslim. Literacy was present in 66.5% (n = 465) and illiteracy in 33.5% (n = 235). By the Modified Kuppuswamy Scale 2021 [20], 50.3% were upper lower, 25.1% lower middle, 15.9% lower, 8.0% upper middle, and 0.7% upper class. The sociodemographic profile of participants is presented in Table 3.

**Table 3:** Sociodemographic profile of study participants (N = 700). The mean age was 39.41 ± 9.2 years.

Characteristic	Category	n	%
Sex	Male	176	25.1
	Female	524	74.9
Age group (years)	30–40	451	64.4
	41–50	148	21.1
	51–60	68	9.7
	61–69	33	4.8
Marital status	Currently married	647	92.4
	Not currently married	53	7.6
Religion	Hindu	499	71.3
	Muslim	194	27.7
	Other	7	1.0
Education	Literate	465	66.5
	Illiterate	235	33.5
Socioeconomic class (Modified Kuppuswamy 2021) [20]	Upper	5	0.7
	Lower middle	176	25.1
	Upper lower	352	50.3
	Lower	111	15.9

Anthropometric and clinical profile

The mean BMI was 23.63 ± 3.64 kg/m². BMI was classified using the WHO international criteria [21]; overweight (BMI 25.0-29.9 kg/m²) was present in 27.1% (n = 190) and obesity (BMI ≥30.0 kg/m²) in 5.6% (n = 39), so that 32.7% (n = 229) were overweight or obese. Abnormal waist-hip ratio was found in 28.6% overall (males, 15.3%; females, 33.0%). Snoring was reported by 38.9% (males, 34.1%; females, 40.5%). Mallampati Class I was present in 77.2%, Class II in 19.4%, and Class III in 3.4%. Physical inactivity was widespread: 80.0% of participants did not meet the WHO threshold of ≥600 MET-minutes per week.

Newly detected hypertension and diabetes

Hypertension was newly detected in 19.3% (n = 135): males 20.5% (n = 36) and females 18.9% (n = 99). The mean systolic blood pressure was 126 ± 17.85 mmHg, and the mean diastolic blood pressure was 80.76 ± 10.84 mmHg. Prevalence rose significantly with age, from 10.2% in the 30-40-year group to 42.4% in those aged 61-69 years (Pearson chi-square test, χ² = 69.12, df = 3, p < 0.001), and varied significantly by socioeconomic status (p = 0.001); the sex difference was not statistically significant (χ² = 0.21, df = 1, p = 0.65). Diabetes was newly detected in 12.3% (n = 86): males 12.5% (n = 22) and females 12.2% (n = 64), a difference that was not statistically significant (χ² = 0.01, df = 1, p = 0.92). The mean fasting blood glucose was 140.1 ± 60.5 mg/dL (median = 121 mg/dL; IQR = 41.75 mg/dL). Prevalence peaked in the 51-60-year group (23.5%) and was lowest in the 30-40-year group (7.8%); no participant in the upper socioeconomic class had newly detected diabetes. The age-group and sex-wise distribution is presented in Table 4.

**Table 4:** Age-group and sex-wise distribution of newly detected hypertension and diabetes (N = 700). Hypertension across age groups was tested with the Pearson chi-square test (χ² = 69.12, df = 3, p < 0.001). Neither hypertension nor diabetes differed significantly between the sexes (hypertension χ² = 0.21, df = 1, p = 0.65; diabetes χ² = 0.01, df = 1, p = 0.92).

Category	Total, n	Hypertension, n (%)	Diabetes, n (%)
Age group (years)			
30–40	451	46 (10.2)	35 (7.8)
41–50	148	49 (33.1)	28 (18.9)
51–60	68	26 (38.2)	16 (23.5)
61–69	33	14 (42.4)	7 (21.2)
Sex			
Male	176	36 (20.5)	22 (12.5)
Female	524	99 (18.9)	64 (12.2)
Total	700	135 (19.3)	86 (12.3)

Prevalence of sleep-related disorders: all tools combined

The integrated point prevalence of all sleep disorders across the four questionnaire instruments is presented in Table 5. By GSAQ, the most frequent disorders were SRBD (37.0%) and primary insomnia (36.0%), followed by insomnia with a mental-disorder component (20.1%), restless legs syndrome (12.0%), parasomnia (8.9%), and periodic limb movement (6.4%). On the ESS, the five-tier distribution was lower normal in 74.3%, higher normal in 20.3%, mild excessive in 2.4%, moderate excessive in 2.1%, and severe excessive in 0.9%, and daytime sleepiness (ESS ≥8) was present in 13.3%. On STOP-Bang, OSA risk was low in 79.1%, intermediate in 17.4%, and high in 3.4%, so that 20.9% screened positive for OSA at the ≥3 cut-off. By PSQI, 23.9% had poor overall sleep quality (global score >5), with poor sleep latency the most common component abnormality (29.7%); 68 participants (9.7%) scored exactly 5 on the global PSQI and were classified as good sleepers.

**Table 5:** Comprehensive prevalence of sleep-related disorders by GSAQ, ESS, STOP-Bang, and PSQI (N = 700). GSAQ = Global Sleep Assessment Questionnaire; ESS = Epworth Sleepiness Scale; OSA = obstructive sleep apnea; PSQI = Pittsburgh Sleep Quality Index

Tool/Disorder	n	%
A. GSAQ – sleep-related disorders (multiple responses per participant permitted)		
Primary insomnia (PI)	252	36.0
Insomnia with mental disorder (IME)	141	20.1
Sleep-related breathing disorder (SRBD)	259	37.0
Restless leg syndrome (RLS)	84	12.0
Periodic limb movement (PLM)	45	6.4
Parasomnia	62	8.9
B. ESS – daytime sleepiness classification		
Lower normal (score 0–5)	520	74.3
Higher normal (score 6–10)	142	20.3
Mild excessive (score 11–12)	17	2.4
Moderate excessive (score 13–15)	15	2.1
Severe excessive (score 16–24)	6	0.9
Daytime sleepiness – EDS (cut-off ≥8)	93	13.3
C. STOP-Bang – OSA risk stratification		
Low risk of OSA (score 0–2)	554	79.1
Intermediate risk of OSA (score 3–4)	122	17.4
High risk of OSA (score 5–8)	24	3.4
OSA positive (cut-off ≥3)	146	20.9
D. PSQI – sleep quality		
Poor sleep latency (>30 minutes to fall asleep)	208	29.7
Short sleep duration (<6 hours/night)	96	13.7
Poor habitual sleep efficiency (<75%)	76	10.9
Poor subjective sleep quality	68	9.7
Daytime dysfunction (PSQI component 7 ≥2)	41	5.9
Sleep disturbances (≥once/week)	39	5.6
Use of sleep medication (any)	30	4.3
Poor overall sleep quality (global PSQI >5)	167	23.9

Sex-stratified prevalence of sleep-related disorders

OSA was markedly more prevalent in males (41.5%) than females (13.9%; p < 0.001), consistent with established anatomical and hormonal differences. Conversely, SRBD (39.9% versus 28.4%, p = 0.008), insomnia with mental disorder (23.1% versus 11.4%, p = 0.001), restless leg syndrome (14.9% versus 3.4%, p < 0.001), periodic limb movement (7.8% versus 2.3%, p = 0.015), and parasomnia (11.3% versus 1.7%, p < 0.001) were all significantly more prevalent in females. These are within-sex proportions and the sex differences are reported as observed; possible explanations are considered in the Discussion as hypotheses rather than as established mechanisms. Primary insomnia, poor sleep quality, hypertension, and diabetes showed no significant sex difference. Sex, together with all other independent variables, is examined against the principal sleep outcomes in Table 6.

**Table 6:** Association of sleep-related disorders with sociodemographic and clinical variables cross-tabulation (N = 700). Data are presented as n (%); statistically significant associations are shown in bold. *: p-value <0.05 was considered statistically significant. ^#^: Fisher’s exact test was used because one or more expected cell frequencies were <5. SRBD was assessed using GSAQ, OSA using STOP-Bang (≥3), and EDS using ESS (≥8). Poor sleep refers to poor overall sleep quality (PSQI >5); Hypertension and diabetes denote newly detected (previously undiagnosed) cases. SRBD = sleep-related breathing disorder; OSA = obstructive sleep apnea; EDS = excessive daytime sleepiness; GSAQ = Global Sleep Assessment Questionnaire; ESS = Epworth Sleepiness Scale; PSQI = Pittsburgh Sleep Quality Index

Category	Total, n	SRBD, n (%)	OSA, n (%)	EDS, n (%)	Poor sleep, n (%)
Sex					
Male	176	50 (28.4)*	73 (41.5)*	28 (15.9)	41 (23.3)
Female	524	209 (39.9)*	73 (13.9)*	65 (12.4)	126 (24.0)
Age group					
30–40 years	451	183 (40.6)	47 (10.4)*	62 (13.7)^#^	112 (24.8)
41–50 years	148	48 (32.4)	42 (28.4)*	21 (14.2)^#^	36 (24.3)
51–60 years	68	18 (26.5)	41 (60.3)*	7 (10.3)^#^	13 (19.1)
61–69 years	33	10 (30.3)	16 (48.5)*	3 (9.1)^#^	6 (18.2)
Body mass index					
<25 kg/m²	471	173 (36.7)	76 (16.1)*	55 (11.7)	121 (25.7)
≥25 kg/m²	229	86 (37.6)	70 (30.6)*	38 (16.6)	46 (20.1)
Waist-hip ratio					
Normal	500	180 (36.0)	112 (22.4)	59 (11.8)	124 (24.8)
Abnormal	200	79 (39.5)	34 (17.0)	34 (17.0)	43 (21.5)
Education					
Illiterate	235	89 (37.9)	48 (20.4)	28 (11.9)	52 (22.1)
Literate	465	170 (36.6)	98 (21.1)	65 (14.0)	115 (24.7)
Socioeconomic status					
Lower	111	40 (36.0)	29 (26.1)	10 (9.0)	29 (26.1)
Upper-lower	352	133 (37.8)	72 (20.5)	46 (13.1)	79 (22.4)
Lower middle	176	66 (37.5)	30 (17.0)	29 (16.5)	46 (26.1)
Upper middle and above	61	20 (32.8)	15 (24.6)	8 (13.1)	13 (21.3)
Physical activity					
Sufficient	140	49 (35.0)	51 (36.4)*	20 (14.3)	38 (27.1)
Insufficient	560	210 (37.5)	95 (17.0)*	73 (13.0)	129 (23.0)
Hypertension status					
Normotensive	565	206 (36.5)	74 (13.1)*	76 (13.5)	142 (25.1)
Hypertensive	135	53 (39.3)	72 (53.3)*	17 (12.6)	25 (18.5)
Diabetes status					
Non-diabetic	614	230 (37.5)	121 (19.7)*	75 (12.2)*	151 (24.6)
Diabetic	86	29 (33.7)	25 (29.1)*	18 (20.9)*	16 (18.6)

Association of Sociodemographic and Cardiovascular Risk Factors With Sleep Disorders

Table 6 presents the cross-tabulation of the four principal sleep outcomes against the sociodemographic and clinical variables examined. All associations in Table 6 were assessed using Pearson’s chi-square test, except for the comparison between age group and EDS, in which one or more expected cell frequencies were below five, and the Fisher exact test was therefore applied; a p-value below 0.05 was considered statistically significant. OSA was significantly associated with sex (χ² = 60.56, df = 1, p < 0.001), age group (χ² = 112.03, df = 3, p < 0.001), BMI (χ² = 19.44, df = 1, p < 0.001), physical activity (χ² = 25.71, df = 1, p < 0.001), newly detected hypertension (χ² = 106.87, df = 1, p < 0.001), and newly detected diabetes (χ² = 4.01, df = 1, p = 0.045), but not with socioeconomic status (χ² = 3.97, df = 3, p = 0.265). SRBD was significantly associated only with sex (χ² = 6.96, df = 1, p = 0.008) and showed a borderline association with age group (χ²=7.67, df=3, p=0.053). EDS was significantly more frequent among participants with diabetes (χ² = 4.97, df = 1, p = 0.026), while poor overall sleep quality showed no significant association with any variable examined (all p > 0.05).​​​​​​​​​​​​​​

Multivariable logistic regression: factors associated with sleep disorders

Each of seven sleep outcomes was regressed on the independent variables showing a significant bivariate association with that outcome, i.e., sex, BMI, waist-hip ratio, physical activity, hypertension and diabetes, in separate binary logistic regression models (Table 7). For STOP-Bang screen positivity, the strongest and most numerous associations were with hypertension (AOR = 9.46, 95% CI = 5.85-15.31), overweight or obesity (BMI ≥25 kg/m²; AOR = 2.43, 95% CI = 1.57-3.76), and male sex (female AOR = 0.19, 95% CI = 0.12-0.31), with screen positivity paradoxically less frequent among physically inactive participants (AOR = 0.45, 95% CI = 0.27-0.74); as noted below, hypertension, BMI, and sex are themselves scoring components of the instrument and these are structural rather than independent associations. Restless legs syndrome was independently associated with female sex (AOR = 4.05) and physical inactivity (AOR = 2.61); insomnia associated with mental disorder (AOR = 2.28) and poor sleep latency (AOR = 1.77) were independently associated with female sex; and daytime sleepiness was independently associated with diabetes (AOR = 1.84) and abnormal waist-hip ratio (AOR = 1.62). Primary insomnia and poor overall sleep quality had no significant independent predictor among the variables examined.

**Table 7:** Multivariable binary logistic regression of seven sleep disorders on sociodemographic and cardiovascular risk factors (N = 700). Each outcome was modelled separately, with variables showing a significant bivariate association entered simultaneously. Bold entries and * denote p-values <0.05. Wald χ² has 1 degree of freedom throughout; model fit statistics are given in the text. Hypertension (blood pressure ≥140/90 mmHg) and diabetes (fasting glucose ≥126 mg/dL) refer to newly detected cases; because male sex, BMI, and hypertension are STOP-Bang scoring components, the OSA estimates are structural associations with screen positivity rather than independent predictors. BMI = body mass index; ESS = Epworth Sleepiness Scale; PSQI = Pittsburgh Sleep Quality Index; OR = odds ratio; CI = confidence interval

Sleep disorder (outcome)	Independent variable	Adjusted OR (95% CI)	Wald χ²	P-value
Primary insomnia	Female sex (ref. male)	1.35 (0.92-1.98)	2.36	0.129
	BMI ≥25 kg/m²	1.20 (0.86-1.68)	1.14	0.272
	Abnormal waist-hip ratio	1.18 (0.83-1.67)	0.86	0.363
	Physical inactivity	0.99 (0.66-1.49)	0.00	0.964
	Hypertension	1.02 (0.68-1.51)	0.01	0.938
	Diabetes	0.63 (0.38-1.05)	3.18	0.078
Insomnia with mental disorder	Female sex (ref. male)	2.28 (1.35-3.86)	9.46	0.002*
	BMI ≥25 kg/m²	1.38 (0.94-2.05)	2.62	0.103
	Abnormal waist-hip ratio	0.79 (0.51-1.21)	1.14	0.271
	Physical inactivity	1.42 (0.83-2.44)	1.62	0.199
	Hypertension	0.95 (0.58-1.54)	0.04	0.832
	Diabetes	0.73 (0.39-1.37)	0.96	0.328
Restless leg syndrome	Female sex (ref. male)	4.05 (1.71-9.59)	10.11	0.002*
	BMI ≥25 kg/m²	1.08 (0.66-1.77)	0.09	0.769
	Abnormal waist-hip ratio	1.18 (0.72-1.94)	0.43	0.518
	Physical inactivity	2.61 (1.09-6.25)	4.64	0.031*
	Hypertension	1.01 (0.55-1.85)	0.00	0.968
	Diabetes	1.07 (0.54-2.13)	0.04	0.847
Daytime sleepiness (ESS ≥8)	Female sex (ref. male)	0.72 (0.43-1.21)	1.55	0.217
	BMI ≥25 kg/m²	1.57 (0.99-2.47)	3.74	0.054
	Abnormal waist-hip ratio	1.62 (1.00-2.62)	3.85	0.049*
	Physical inactivity	0.83 (0.47-1.47)	0.41	0.519
	Hypertension	0.83 (0.46-1.48)	0.39	0.518
	Diabetes	1.84 (1.01-3.35)	3.97	0.045*
Obstructive sleep apnea (STOP-Bang ≥3)	Female sex (ref. male)	0.19 (0.12-0.31)	47.05	<0.001*
	BMI ≥25 kg/m²	2.43 (1.57-3.76)	15.88	<0.001*
	Abnormal waist-hip ratio	1.11 (0.67-1.86)	0.16	0.682
	Physical inactivity	0.45 (0.27-0.74)	9.64	0.002*
	Hypertension	9.46 (5.85-15.31)	83.83	<0.001*
	Diabetes	1.52 (0.83-2.78)	1.84	0.180
Poor sleep latency	Female sex (ref. male)	1.77 (1.16-2.70)	7.02	0.008*
	BMI ≥25 kg/m²	0.79 (0.56-1.14)	1.69	0.207
	Abnormal waist-hip ratio	0.94 (0.65-1.37)	0.11	0.757
	Physical inactivity	0.95 (0.62-1.47)	0.05	0.830
	Hypertension	0.77 (0.50-1.19)	1.40	0.246
	Diabetes	1.03 (0.62-1.72)	0.01	0.905
Poor overall sleep quality (PSQI >5)	Female sex (ref. male)	1.12 (0.73-1.71)	0.27	0.609
	BMI ≥25 kg/m²	0.74 (0.50-1.09)	2.29	0.124
	Abnormal waist-hip ratio	0.82 (0.55-1.24)	0.92	0.348
	Physical inactivity	0.81 (0.52-1.27)	0.86	0.356
	Hypertension	0.70 (0.43-1.12)	2.13	0.139
	Diabetes	0.79 (0.44-1.43)	0.61	0.439

Because hypertension, BMI, and male sex are themselves scoring components of the STOP-Bang instrument, the OSA associations are partly structural and must be interpreted with this in mind. Two points are relevant to the hypertension component in particular. First, all participants with previously known hypertension were excluded at enrolment, so every participant entered the study reporting no history of hypertension and would have answered the STOP-Bang blood pressure item in the negative at the point of questionnaire administration; the item was scored positive only where the study measurement itself identified raised blood pressure by the JNC 8 threshold [23]. Second, this is also the reason participants with known hypertension, diabetes, and other chronic disease were excluded at the outset. The estimates for hypertension, BMI, and sex in this model are therefore reported as structural associations with STOP-Bang screen positivity and not as independent epidemiological predictors of OSA.

Model fit statistics for the seven models were as follows. Discrimination and explained variation were modest for most outcomes and substantial only for STOP-Bang screen positivity: Nagelkerke R² was 0.015 for primary insomnia, 0.043 for insomnia with mental disorder, 0.073 for restless legs syndrome, 0.034 for daytime sleepiness, 0.354 for STOP-Bang screen positivity, 0.022 for poor sleep latency and 0.017 for poor overall sleep quality. The Hosmer-Lemeshow goodness-of-fit test (df = 8) was non-significant for six of the seven models, indicating adequate calibration: primary insomnia (χ² = 7.59, p = 0.474), insomnia with mental disorder (χ² = 6.13, p = 0.633), restless legs syndrome (χ² = 3.87, p = 0.869), daytime sleepiness (χ² = 3.82, p = 0.873), STOP-Bang screen positivity (χ² = 9.64, p = 0.291), and poor sleep latency (χ² = 11.31, p = 0.185), but was significant for poor overall sleep quality (χ² = 20.79, p = 0.008), indicating that this model was poorly calibrated and that its estimates, none of which reached significance, should be interpreted with corresponding caution. The area under the receiver operating characteristic curve was 0.835 for STOP-Bang screen positivity and between 0.57 and 0.64 for the remaining six outcomes, so that apart from the STOP-Bang model, which is in any case structurally determined by its own scoring components, the variables examined discriminated poorly between participants with and without each sleep outcome.

**Figure 2:**
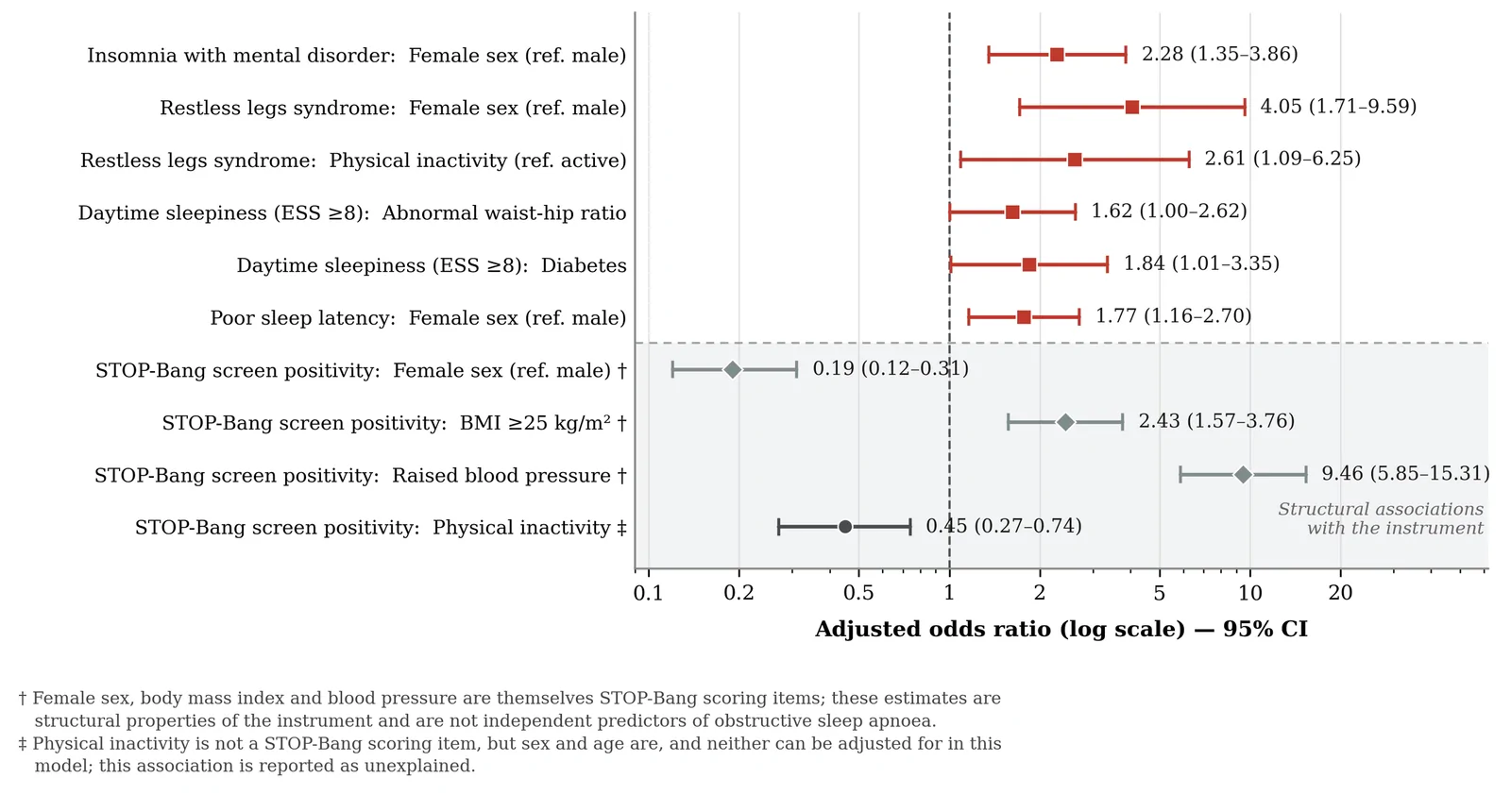
Statistically significant adjusted odds ratios from multivariable logistic regression (N = 700). Estimates plotted on a logarithmic scale with 95% confidence intervals. Adjusted associations (unshaded): insomnia with mental disorder, female sex 2.28 (1.35–3.86); restless leg syndrome, female sex 4.05 (1.71–9.59), and physical inactivity 2.61 (1.09–6.25); daytime sleepiness (ESS ≥8), abnormal waist-hip ratio 1.62 (1.00–2.62), and diabetes 1.84 (1.01–3.35); poor sleep latency, female sex 1.77 (1.16–2.70). Estimates for STOP-Bang screen positivity (shaded): female sex 0.19 (0.12–0.31), BMI ≥25 kg/m² 2.43 (1.57–3.76), raised blood pressure 9.46 (5.85–15.31), and physical inactivity 0.45 (0.27–0.74). ^†^ Female sex, BMI, and blood pressure are themselves STOP-Bang scoring items, so these three estimates are structural properties of the instrument and are not independent predictors of obstructive sleep apnoea. ^‡^ Physical inactivity is not a scoring item, but sex and age are, and neither can be adjusted for in this model, so that association is reported as unexplained. BMI = body mass index; ESS = Epworth Sleepiness Scale

Figure 2 displays all statistically significant AORs across the seven models, showing the dominant association of hypertension with OSA, the consistent female predominance of insomnia-related and restless legs symptoms, and the contrasting male predominance of OSA.

## Discussion

This community-based study provides prevalence estimates for selected SRDs, assessed by four validated screening questionnaires, in an urban adult population of South Delhi. The findings reveal a high burden of SRBD (37.0%) and primary insomnia (36.0%) by GSAQ. Recent polysomnographic data from the Indian BLESS cohort reported moderate-to-severe OSA in 30.5% of adults [32], confirming that sleep-disordered breathing is far more common in India than older estimates suggested; the high GSAQ-based SRBD figure observed here is consistent with this and reflects the questionnaire’s broad symptom-based sensitivity rather than polysomnographic specificity.

The STOP-Bang screen-positive OSA prevalence of 20.9% (intermediate-or-high risk; cut-off ≥3) is consistent with the substantial sleep-disordered breathing burden reported in recent polysomnographic estimates from Indian and European populations [32,33], while remaining a screening rather than a diagnostic figure. OSA risk was markedly higher in males (41.5%) than females (13.9%; p < 0.001) and rose steeply with age, in keeping with population-based determinants of OSA reported internationally [33]. With intermediate risk in 17.4% and high risk in 3.4%, roughly one in five apparently healthy urban adults carries a clinically meaningful OSA risk profile.

Daytime sleepiness (ESS ≥8) was present in 13.3%. The five-tier ESS classification adds important clinical granularity beyond a simple binary cut-off: the 5.4% with mild-to-severe EDS (score ≥11) require clinical evaluation, whereas the 20.3% with higher-normal sleepiness represent a substantial prevention group. This distinction has particular relevance for occupational safety and road traffic risk in a working-age urban population.

Poor overall sleep quality (PSQI >5) affected 23.9% of participants, lower than the high rates reported among other urban Indian populations such as slum-dwelling adults [34]. The most prevalent PSQI abnormality was poor sleep latency (29.7%), indicating that difficulty initiating sleep is the predominant specific complaint in this population, with direct implications for behavioral sleep-hygiene interventions.

Contrary to the classic OSA risk profile, neither obesity, central adiposity, nor older age was associated with GSAQ-defined SRBD (all p > 0.05); the only significant correlate was female sex (Pearson chi-square test, χ² = 6.96, df = 1, p = 0.008). This divergence is informative. The GSAQ defines SRBD from self-reported loud snoring and witnessed breathing pauses and has modest specificity (58%) [17], capturing a broad symptom-based construct that overlaps only partly with polysomnographically defined OSA. Women with sleep-disordered breathing characteristically report non-specific symptoms such as insomnia, fatigue, and unrefreshing sleep rather than the classic snoring and witnessed apnea picture, and are consequently underrecognized when male-derived criteria are applied [35,36]. The higher SRBD symptom reporting among women in this sample, despite their substantially lower OSA risk on STOP-Bang, is consistent with these sex differences in symptom expression and underscores that questionnaire-defined SRBD and instrument-scored OSA should not be treated as interchangeable. By contrast, OSA risk did follow the expected pattern, being associated with male sex, older age, and obesity, although the STOP-Bang score incorporates several of these characteristics as scoring items, which limits independent causal inference.

Newly detected hypertension in 19.3% of ostensibly healthy participants, rising from 10.2% at 30-40 years to 42.4% at 61-69 years, together with newly detected diabetes in 12.3%, documents a substantial silent cardiometabolic burden. Within this cohort, OSA was strongly associated with newly detected hypertension (53.3% versus 13.1% in normotensives; p < 0.001) and with newly detected diabetes (29.1% versus 19.7%; p = 0.045), consistent with the separately reported OSA analysis of this population [37]; by contrast, GSAQ-defined SRBD, daytime sleepiness, and poor sleep quality were not associated with either hypertension or diabetes (all p > 0.05), indicating that questionnaire-based sleep symptoms and instrument-screened OSA capture different cardiometabolic risk. In the Indian BLESS cohort, polysomnographic OSA was independently associated with diabetes (odds ratio = 2.14), hypertension (odds ratio = 1.98), and metabolic syndrome (odds ratio = 2.36) [32], underscoring the clinical value of identifying sleep-disordered breathing alongside cardiometabolic screening in apparently healthy adults.

Notably, the conventional obesity-age-sex axis explained little of the variation in GSAQ-defined SRBD, implying that its determinants in this community extend beyond anthropometry to socioeconomic and behavioural factors; socioeconomic position, in particular, is increasingly recognized as a determinant of sleep health [38]. Unmeasured contributors such as nasal pathology, sleep position, alcohol use, psychological distress, and environmental noise are also likely relevant. Physical inactivity, affecting 80.0% of participants, was associated with restless leg syndrome, and the high prevalence of inactivity in this population is itself notable [19]. The relationship between physical activity and STOP-Bang-defined OSA ran counter to expectation: OSA risk was higher among the sufficiently active (36.4%) than the inactive (17.0%) (p < 0.001), whereas randomized evidence shows that exercise training reduces apnea severity [39]. We do not offer a tested explanation for this observation. The GPAQ measures physical activity across work, travel, and recreation domains and therefore already incorporates occupational activity within the total MET-minutes score; occupation was consequently not entered separately into the regression models, as it would have duplicated information already captured by the GPAQ. Detailed occupational profiles of individual participants were not recorded, only the occupation of the head of the household, as a component of the Modified Kuppuswamy scale, so the contribution of occupational activity to this finding could not be examined directly. The observation is therefore reported as unexplained and potentially attributable to residual confounding, selection, measurement limitations of the GPAQ, or chance, and this is acknowledged among the limitations below.

Strengths and limitations

Strengths include the community-based design, the large probability sample (n = 700), the use of four complementary validated instruments, four-stage random sampling with ASHA register-based household enumeration, standardized clinical detection of newly detected hypertension and diabetes, sex-stratified and cross-tabulated reporting of all outcomes, and the administration of every instrument by a single trained interviewer, which eliminates inter-interviewer variation.

The cross-sectional design precludes any inference about temporal sequence or causation. All sleep outcomes rest on questionnaire-based screening rather than diagnostic assessment: the four instruments are pre-validated and were pre-tested in this population, but none is a diagnostic gold standard, and polysomnography, the reference standard for sleep-disordered breathing, was not performed. The reported sensitivity and specificity of each instrument are given in the Materials and Methods section, and several GSAQ constructs have modest specificity (49-80%), so that in a community setting with low underlying prevalence, a substantial proportion of screen-positive results will be false positives; the prevalence figures reported here should therefore be read as instrument-defined screening positivity rather than as the prevalence of clinically diagnosed disorders. Eligibility rested on self-reported history alone, without corroboration from medical records or prescriptions, so participants with undiagnosed or unreported chronic disease may have been included. Hypertension was ascertained from three readings taken within a single sitting rather than on separate occasions, which may overestimate its prevalence through the white-coat effect and regression to the mean. Age was not entered into the multivariable models because it was not significantly associated with the majority of outcomes on bivariate testing; for STOP-Bang positivity, where age was significantly associated, age is itself a scoring component of the instrument, so its inclusion would have compounded the structural circularity already present in that model, and residual confounding by age cannot be excluded. For the same reason, sex, BMI, and blood pressure are themselves STOP-Bang scoring items and could not be removed from that model without removing the outcome’s own components; the associations reported for them are structural and are labelled as such. Detailed occupational profiles were not recorded, which prevented direct examination of the unexpected physical activity finding. The higher female proportion (74.9%) arising from daytime data collection affects the pooled prevalence estimates, weighting upward those constructs more frequent among women and downward those more frequent among men, although the within-sex proportions reported here are unaffected. The study area also represents only the Central Zone of the SDMC, limiting generalizability to other zones or to non-urban populations.

## Conclusions

SRDs are highly prevalent in the adult urban population of South Delhi, with SRBD, primary insomnia, poor sleep quality, daytime sleepiness, and OSA all common, confirming that the burden of sleep pathology in this community extends well beyond OSA alone and spans the symptom domains captured by the four instruments used. STOP-Bang screen positivity was concentrated among men and older participants and among those with a higher BMI, whereas GSAQ-defined SRBD was more frequent in women and was not explained by anthropometric or demographic factors, indicating that the two measures capture different, only partly overlapping, aspects of sleep-disordered breathing rather than being interchangeable proxies for the same underlying condition. On multivariable analysis, STOP-Bang screen positivity was most strongly associated with hypertension, overweight or obesity, and male sex; because all three are scoring components of the instrument, these are structural associations with screen positivity and are not interpretable as independent predictors of OSA. In contrast, restless legs syndrome and insomnia-related outcomes were independently linked to female sex, and daytime sleepiness tracked with diabetes and abnormal waist-hip ratio, underscoring that different sleep phenotypes in this population are driven by distinct, sex- and metabolically patterned risk pathways rather than a single common mechanism. Newly detected hypertension and diabetes in participants who were apparently healthy at enrolment document a substantial, coexisting silent cardiometabolic burden that would otherwise have gone unrecognized outside a community screening setting. These findings support two observations. First, questionnaire-based screening using the GSAQ, ESS, STOP-Bang and PSQI is inexpensive, scalable, and well suited to integration into primary healthcare and non-communicable disease control programs, where it can flag both sleep-related symptoms and previously undetected hypertension or diabetes within the same community encounter. Second, physical inactivity was highly prevalent in this population and was independently associated with restless leg syndrome. As a cross-sectional screening survey, however, this study cannot establish temporal sequence, causation, or intervention effectiveness, and no inference is drawn that promoting physical activity or weight reduction would alter any of the sleep outcomes examined; the inverse association observed between physical inactivity and STOP-Bang positivity underlines that caution. Longitudinal studies with objective sleep assessment and interventional evaluation would be required to address those questions. Given the sex-patterned and instrument-specific nature of the associations observed, future community surveillance should stratify by sex and by diagnostic tool rather than treating sleep-disordered breathing as a single, uniform entity.

## Figures and Tables

**Figure 3:**
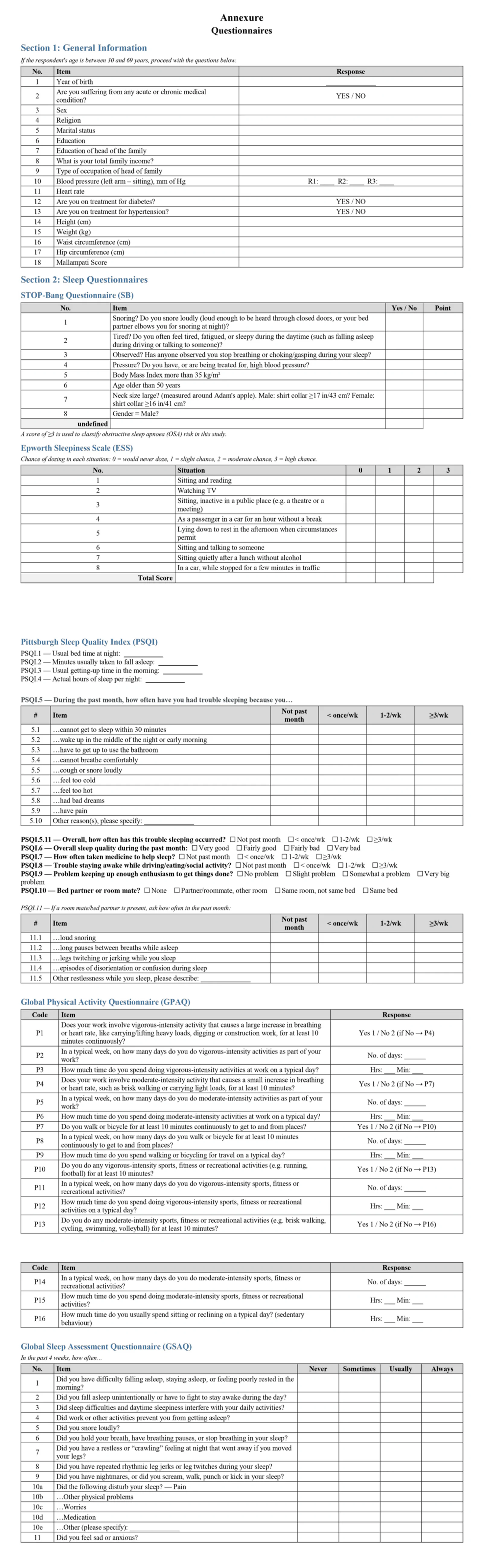
Study questionnaires.

## References

[1] (2026). American Academy of Sleep Medicine. International Classification of Sleep Disorders (ICSD). https://aasm.org/clinical-resources/international-classification-sleep-disorders/.

[2] Ohayon MM (2002). Epidemiology of insomnia: what we know and what we still need to learn. Sleep Med Rev.

[3] Suri JC, Sen MK, Adhikari T (2008). Epidemiology of sleep disorders in the adult population of Delhi: a questionnaire based study. Indian J Sleep Med.

[4] Panda S, Taly AB, Sinha S, Gururaj G, Girish N, Nagaraja D (2012). Sleep-related disorders among a healthy population in South India. Neurol India.

[5] World Health Organization (2016). International Statistical Classification of Diseases and Related Health Problems 10th Revision. https://icd.who.int/browse10/2016/en.

[6] Senaratna CV, Perret JL, Lodge CJ (2017). Prevalence of obstructive sleep apnea in the general population: a systematic review. Sleep Med Rev.

[7] Pattanaik S, Rajagopal R, Mohanty N (2018). Prevalence of obstructive sleep apnea in an Indian population: using STOP-Bang questionnaire. Asian J Pharm Clin Res.

[8] Jean-Louis G, Zizi F, Clark LT, Brown CD, McFarlane SI (2008). Obstructive sleep apnea and cardiovascular disease: role of the metabolic syndrome and its components. J Clin Sleep Med.

[9] Pahwa P, Karunanayake CP, Hagel L, Gjevre JA, Rennie D, Lawson J, Dosman JA (2012). Prevalence of High Epworth Sleepiness Scale scores in a rural population. Can Respir J.

[10] Reddy EV, Kadhiravan T, Mishra HK, Sreenivas V, Handa KK, Sinha S, Sharma SK (2009). Prevalence and risk factors of obstructive sleep apnea among middle-aged urban Indians: a community-based study. Sleep Med.

[11] Basnet S, Merikanto I, Lahti T, Männistö S, Laatikainen T, Vartiainen E, Partonen T (2016). Associations of common chronic non-communicable diseases and medical conditions with sleep-related problems in a population-based health examination study. Sleep Sci.

[12] Trakada G, Chrousos G, Pejovic S, Vgontzas A (2007). Sleep apnea and its association with the stress system, inflammation, insulin resistance and visceral obesity. Sleep Med Clin.

[13] Tasali E, Mokhlesi B, Van Cauter E (2008). Obstructive sleep apnea and type 2 diabetes: interacting epidemics. Chest.

[14] Montag SE, Knutson KL, Zee PC, Goldberger JJ, Ng J, Kim KA, Carnethon MR (2017). Association of sleep characteristics with cardiovascular and metabolic risk factors in a population sample: the Chicago Area Sleep Study. Sleep Health.

[15] Drager LF, Togeiro SM, Polotsky VY, Lorenzi-Filho G (2013). Obstructive sleep apnea: a cardiometabolic risk in obesity and the metabolic syndrome. J Am Coll Cardiol.

[16] Mullington JM, Haack M, Toth M, Serrador JM, Meier-Ewert HK (2009). Cardiovascular, inflammatory, and metabolic consequences of sleep deprivation. Prog Cardiovasc Dis.

[17] Roth T, Zammit G, Kushida C, Doghramji K, Mathias SD, Wong JM, Buysse DJ (2002). A new questionnaire to detect sleep disorders. Sleep Med.

[18] Buysse DJ, Reynolds CF 3rd, Monk TH, Berman SR, Kupfer DJ (1989). The Pittsburgh Sleep Quality Index: a new instrument for psychiatric practice and research. Psychiatry Res.

[19] (2026). Global Physical Activity Questionnaire (GPAQ) Analysis Guide. https://www.who.int/docs/default-source/ncds/ncd-surveillance/gpaq-analysis-guide.pdf..

[20] Saleem SM, Jan SS (2021). Modified Kuppuswamy socioeconomic scale updated for the year 2021. Indian J Forensic Community Med.

[21] World Health Organization (2000). Obesity: Preventing and Managing the Global Epidemic. Report of a WHO Consultation. https://iris.who.int/handle/10665/42330.

[22] Chobanian AV, Bakris GL, Black HR (2003). The Seventh Report of the Joint National Committee on Prevention, Detection, Evaluation, and Treatment of High Blood Pressure: the JNC 7 report. JAMA.

[23] James PA, Oparil S, Carter BL (2014). 2014 evidence-based guideline for the management of high blood pressure in adults: report from the panel members appointed to the Eighth Joint National Committee (JNC 8). JAMA.

[24] (2026). ICMR Guidelines for Management of Type 2 Diabetes 2018. Diabetes.

[25] (2026). Diabetes tests and diagnosis. https://www.niddk.nih.gov/health-information/diabetes/overview/tests-diagnosis.

[26] Bajpai G, Shukla G, Pandey RM (2016). Validation of a modified Hindi version of the Epworth Sleepiness Scale among a North Indian population. Ann Indian Acad Neurol.

[27] Johns MW (1991). A new method for measuring daytime sleepiness: the Epworth sleepiness scale. Sleep.

[28] Chung F, Subramanyam R, Liao P, Sasaki E, Shapiro C, Sun Y (2012). High STOP-Bang score indicates a high probability of obstructive sleep apnoea. Br J Anaesth.

[29] Chung F, Abdullah HR, Liao P (2016). STOP-Bang questionnaire: a practical approach to screen for obstructive sleep apnea. Chest.

[30] Bull FC, Maslin TS, Armstrong T (2009). Global physical activity questionnaire (GPAQ): nine country reliability and validity study. J Phys Act Health.

[31] (2026). Epicollect5 data collection tool. https://five.epicollect.net/.

[32] Goyal A, Pakhare A, Pavirala ST (2025). Prevalence and association analysis of obstructive sleep apnea in India: results from BLESS cohort. Sleep Med.

[33] Balagny P, Vidal-Petiot E, Renuy A (2023). Prevalence, treatment and determinants of obstructive sleep apnoea and its symptoms in a population-based French cohort. ERJ Open Res.

[34] Rao M, Bhushan S, Ts R (2023). Poor quality of sleep and it's associated factors among urban slum-dwellers of Bengaluru- a cross-sectional study. F1000Res.

[35] Bouloukaki I, Tsiligianni I, Schiza S (2021). Evaluation of obstructive sleep apnea in female patients in primary care: time for improvement?. Med Princ Pract.

[36] Sodhi A, Pisani M, Glassberg MK, Bourjeily G, D'Ambrosio C (2022). Sex and gender in lung disease and sleep disorders: a state-of-the-art review. Chest.

[37] Singh VK, Islam F, Pathak R, Shah A (2025). Obstructive sleep apnoea in adult population in an urban area of Delhi, India: a community-based epidemiological study. Prev Med Res Rev.

[38] Papadopoulos D, Etindele Sosso FA (2023). Socioeconomic status and sleep health: a narrative synthesis of 3 decades of empirical research. J Clin Sleep Med.

[39] Iftikhar IH, Kline CE, Youngstedt SD (2014). Effects of exercise training on sleep apnea: a meta-analysis. Lung.

